# Analysis of renal functions and proteinuria in young obese adults

**DOI:** 10.1007/s40618-015-0272-0

**Published:** 2015-04-02

**Authors:** D.-Y. You, Z.-Y. Wu, J.-X. Wan, J. Cui, Z.-H. Zou

**Affiliations:** Department of Nephrology, The First Affiliated Hospital of Fujian Medical University, Fuzhou, 350005 China

**Keywords:** Obesity, Young adults, Proteinuria, Renal function, Chronic kidney disease

## Abstract

**Objective:**

To investigate the prevalence of obesity in young adults and to analyze the influencing factors on renal functions and proteinuria in this population.

**Methods:**

This study comprised civil servants between 20 and 39 years old, who received physical examinations at the First Affiliated Hospital of Fujian Medical University. The subjects were categorized into four groups based on age (20–24, 25–29, 30–34 and 35–39 years) and the number of risk factors they had (hypertension, dyslipidemia, hyperglycemia and hyperuricemia). The relationships between obesity and the prevalence of proteinuria, between obesity and risk factors and between estimated glomerular filtration rate (eGFR) and proteinuria were analyzed.

**Results:**

Among the 2293 young civil servants, in men the prevalence of obesity was 33.3 % and proteinuria was 2.5 %. However in women the prevalence of obesity and proteinuria was 7.5 % and 1.7 %, respectively. The levels of blood pressure, serum uric acid (UA), cholesterol (TC), triglyceride (TG), fasting glucose (FBG) and low-density lipoprotein cholesterol (LDL-C) were lower and the level of serum high-density lipoprotein cholesterol (HDL-C) was higher in nonobese groups compared with obese groups. There were no significant differences in eGFR between the two groups. The eGFR in male subjects was associated with age, UA, body mass index (BMI), FBG, TC, TG, LDL and HDL, and in female subjects associated with UA, age, BMI, diastolic blood pressure, FBG and LDL. BMI in both males and females increased with the higher number of risk factors. Multiple regression analysis revealed that hypertension, dyslipidemia, hyperglycemia and hyperuricemia were independently associated with obesity. eGFR decreased with a higher number of risk factors. Obesity, blood pressure, dyslipidemia, hyperglycemia and hyperuricemia were independently associated with proteinuria.

**Conclusion:**

Obesity can pose an independent risk factor for proteinuria in young adults. Hypertension, dyslipidemia, hyperglycemia and hyperuricemia were independently associated with obesity. eGFR decreased with a higher number of risk factors.

## Introduction

Obesity has become a global epidemic and its prevalence in adult population is increasing at a rapid pace. Obesity, along with hypertension, impaired glucose tolerance or diabetes mellitus and dyslipidemia are well-known factors of metabolic syndrome. Together, they increase the risk of cardiovascular disorders and chronic kidney disease (CKD). Currently, kidney damages attributed to obesity are getting more attention. The morbidity of obesity-related glomerulopathy (ORG) increases with obesity. In the USA, the morbidity of ORG was 0.2 % in 1986–1990, while it increased to about 2 % in 1996–2000 [1]. Therefore, it is crucial that renal damage in young obese adults should be analyzed at the early stage. The aim of this study was to compare the clinical characteristics of obese vs. nonobese young adult subjects and to analyze the factors that influence decreased renal function and proteinuria in young obese adults.

## Subjects and methods

### Study population

The subjects were young civil servants between 20 and 39 years old in Fuzhou City. They received physical examination at the First Affiliated Hospital of Fujian Medical University between November 2008 and August 2009. Subjects who failed to provide data such as routine urine test and serum creatinine were excluded from the study. Also, of the subjects who received repeated examinations, only the first examination results were included.

### Measurements

#### Physical examination

The height, body weight, body mass index (BMI), systolic blood pressure (SBP) and diastolic blood pressure (DBP) of all subjects were measured according to the unified standards. The BMI of each subject was calculated by dividing the weight (kg) by the square of the height (m^2^). The blood pressure was measured using an electrical manometer after letting the subjects rest for 15 min. Blood pressure measurement was performed up to three times according to JNCVII standard, and the mean value was used for statistical analysis. The interval between each measurement was 5 min. In case there was a difference of more than 10 mmHg between two measurements; we chose the two closest measurements to calculate the mean value. The subject’s height was defined as net height measured without shoes. Body weight was measured with empty stomach and no coat.

#### Laboratory tests

For laboratory analysis, subjects’ morning urine samples were collected. Female subjects who were menstruating were excluded. Serum creatinine (Scr), urea nitrogen (BUN), uric acid (UA), fasting glucose (FBG), total cholesterol (TC), triglycerides (TG), high-density lipoprotein cholesterol (HDL-C) and low-density lipoprotein cholesterol (LDL-C) were tested. Urinary protein and occult blood were measured by urine dipstick test. Urine red cell counting was carried out by an automatic analyzer (uFl00, Sysmex, Japan). Proteinuria ≥0.3 g/L was considered positive. FBG was detected by hexokinase method with an intra-assay coefficient of variation <2.0 % and inter-assay coefficient of variation <2.5 %. FBG ≥ 6.1 mmol/L was considered abnormal. TC, TG, HDL-C, LDL-C, UA and Scr were measured using a full automatic biochemical analyzer (Hitachi 7600) with an intra-assay coefficient of variation <1.5 % and inter-assay coefficient of variation <2.5 %.

#### Diagnostic code

Estimated glomerular filtration rate (eGFR) was calculated using the equation of MDRD as follows: 186 × Scr(mg/dl) ^ (−1.154) × age ^ (−0.203), including a correction factor of 0.742 for women [2]. 1 µmol Scr = 0.0113 mg/dl.

##### The diagnosis of metabolic syndrome (Chinese Diabetes Society, 2004)

(1) Overweight or obesity was defined as BMI ≥ 25.0 kg/m^2^. (2) Dyslipidemia was defined as TG ≥ 1.70 mmol/L and/or HDL-C < 0.9 mmol/L (men) and  < 1.0 mmol/L (women). (3) Hypertension was defined as SBP ≥ 140 mmHg and/or DBP ≥ 90 mmHg, including those who had been diagnosed with hypertension and had received treatment. (4) Hyperglycemia was defined as FBG ≥ 6.1 mmol/L and 2 h postprandial blood glucose ≥ 11.1 mmol/L, and/or those who had been diagnosed with diabetes. (5) Hyperuricemia was defined as UA ≥ 420 μmol/L (men) and ≥ 360 μmol/L (women). Based on the number of CKD risk factors (dyslipidemia, hypertension, hyperglycemia and hyperuricemia), the subjects were classified into group 0 (no risk factor), group 1 (one risk factor), group 2 (two risk factors) and group 3 (three or four risk factors).

### Statistical analysis

All the data were entered into Excel by two researchers. The data were presented as *x* ± *s*. Student’s *t* test was used to evaluate differences between the obese and nonobese groups. The enumeration data were analyzed by *χ* ^2^ test. Related risk factors that affect renal function were analyzed by multivariate regression analysis and stepwise regression analysis with variables including age, BMI, SBP, DBP, FBG, TC, TG, LDL-C, HDL-C and UA. Related risk factors for proteinuria were analyzed by two classifications of non-conditional logistic regression analysis with variables including gender, age, BMI, obesity, hypertension, dyslipidemia, high FBG and hyperuricemia. All data were analyzed using SPSS version 11.7.

## Results

### Subjects’ baseline characteristics and physical examination data

A total of 1292 male and 1001 female eligible subjects were enrolled in our study. Their baseline characteristics are represented in Table 1. 33.3 % of male and 7.5 % of female subjects were obese. The gender differences in the characteristics of obesity and nonobesity in different age groups and in the same age group are shown in Tables 2 and 3. The average age was similar between obese and nonobese subjects in different age groups. In male subjects of different ages, blood pressure, TC, TG and LDL-C were higher, while HDL-C level was lower in the obese group compared with the nonobese group. UA in the 25- to 29-year age group, 30- to 34-year age group and 35- to 39-year age group was higher in the obese group than in the nonobese group. FBG in the 30- to 34-year age group and in the 35- to 39-year age group was higher in the obese group than in the nonobese group. In female subjects, HDL-C in the 25- to 29-year age group, 30- to 34-year age group and 35- to 39-year age group was lower in the obese group than in the nonobese group. TG and UA in the 30- to 34-year age group and in the 35- to 39-year age group was higher in the obese group than in the nonobese group. eGFR was similar in male and female subjects of different age groups.

**Table 1:** Characteristics of males and females analyzed

Characteristics	Male	Female	*P* value
*n*	1292	1001	–
Age (years)	31.8 ± 5.5	31.3 ± 5.3	0.042
Body mass index (kg/m^2^)	23.5 ± 3.3	21.1 ± 2.6	0.000
Obesity (%)	33.3	7.5	0.000
Systolic BP (mm Hg)	114.3 ± 13.4	106.7 ± 11.8	0.000
Diastolic BP (mm Hg)	75.7 ± 9.8	69.9 ± 8.7	0.000
Proteinuria ( %)	2.5	1.7	0.000
Serum creatinine (μmol/L)	75.2 ± 9.8	51.7 ± 7.3	0.000
eGFR (ml/min)	113.9 ± 17.8	131.2 ± 21.7	0.000
FBG (mmol/L)	5.0 ± 0.7	4.8 ± 0.4	0.000
TC (mmol/L)	4.88 ± 0.91	4.60 ± 0.82	0.000
TG (mmol/L)	1.54 ± 1.03	0.87 ± 0.45	0.000
LDL-C (mmol/L)	2.80 ± 0.76	2.44 ± 0.68	0.000
HDL-C (mmol/L)	1.32 ± 0.28	1.60 ± 0.34	0.000
UA (mmol/L)	383.6 ± 69.3	271.5 ± 51.5	0.000

**Table 2:** Characteristics of male obese and nonobese subjects

Characteristics	20–24 years			25–29 years		
	Obese	Nonobese	*P* value	Obese	Nonobese	*P* value
*N*	31	126	–	88	179	–
Age (years)	22.6 ± 1.3	22.3 ± 1.3	0.160	27.1 ± 1.5	26.7 ± 1.4	0.488
Body mass index (kg/m^2^)	27.1 ± 2.4	20.7 ± 2.1	0.000	27.2 ± 2.3	21.5 ± 2.1	0.000
Systolic BP (mm Hg)	120.6 ± 20.3	114.3 ± 13.4	0.037	115.9 ± 13.2	114.3 ± 12.1	0.312
Diastolic BP (mm Hg)	77.6 ± 11.4	72.8 ± 8.3	0.008	77.3 ± 10.1	73.3 ± 8.8	0.001
Proteinuria (%)	0	4.5	0.000	3.4	2.2	0.572
Serum creatinine (μmol/L)	76.7 ± 10.3	75.0 ± 8.3	0.323	77.7 ± 9.6	75.7 ± 9.0	0.100
eGFR (ml/min)	119.1 ± 19.0	121.9 ± 16.2	0.408	112.7 ± 16.3	116.1 ± 17.0	0.119
FBG (mmol/L)	5.1 ± 0.7	4.9 ± 0.6	0.206	4.9 ± 0.6	4.9 ± 0.4	0.686
TC (mmol/L)	4.73 ± 0.70	4.44 ± 0.78	0.053	5.34 ± 0.93	4.63 ± 0.85	0.000
TG (mmol/L)	1.33 ± 0.68	1.07 ± 0.58	0.038	2.11 ± 1.35	1.24 ± 0.74	0.000
LDL (mmol/L)	2.86 ± 0.59	2.52 ± 0.66	0.011	3.19 ± 0.79	2.57 ± 0.73	0.000
HDL (mmol/L)	1.19 ± 0.17	1.38 ± 0.29	0.002	1.25 ± 0.25	1.37 ± 0.29	0.001
UA (mmol/L)	402.7 ± 79.3	378.9 ± 71.1	0.105	409.2 ± 71.6	371.6 ± 63.8	0.000

Characteristics	30–34 years			35–39 years		
	Obese	Nonobese	*P* value	Obese	Nonobese	*P* value
*N*	126	241	–	185	316	–
Age (years)	32.3 ± 1.4	32.1 ± 1.4	0.264	37.1 ± 1.4	37.1 ± 1.4	0.856
Body mass index (kg/m^2^)	27.1 ± 2.8	21.9 ± 2.0	0.000	27.0 ± 1.6	22.1 ± 1.9	0.000
Systolic BP (mm Hg)	115.1 ± 11.7	112.0 ± 12.0	0.009	118.7 ± 15.0	112.1 ± 13.1	0.000
Diastolic BP (mmHg)	78.3 ± 9.7	73.8 ± 8.9	0.000	81.6 ± 11.3	75.1 ± 9.3	0.000
Proteinuria (%)	4.8	1.2	0.068	4.3	0.6	0.007
Serum creatinine (μmol/L)	75.9 ± 11.1	76.0 ± 9.2	0.831	75.3 ± 10.4	73.2 ± 10.1	0.035
eGFR (ml/min)	112.7 ± 19.0	114.5 ± 15.4	0.515	110.3 ± 17.8	113.8 ± 19.0	0.039
FBG (mmol/L)	5.2 ± 0.7	4.9 ± 0.5	0.000	5.3 ± 1.3	5.0 ± 0.7	0.000
TC (mmol/L)	5.10 ± 1.14	4.82 ± 0.85	0.008	5.23 ± 0.87	4.82 ± 0.82	0.000
TG (mmol/L)	2.03 ± 1.35	1.33 ± 0.94	0.000	2.11 ± 1.21	1.41 ± 0.77	0.000
LDL (mmol/L)	2.97 ± 0.92	2.74 ± 0.72	0.010	3.07 ± 0.74	2.74 ± 0.68	0.000
HDL (mmol/L)	1.22 ± 0.21	1.35 ± 0.27	0.000	1.21 ± 0.23	1.36 ± 0.30	0.000
UA (mmol/L)	402.6 ± 71.0	373.8 ± 66.4	0.000	395.9 ± 69.7	376.1 ± 67.3	0.009

**Table 3:** Characteristics of female obese and nonobese subjects

Characteristics	20–24 years			25–29 years		
	Obese	Nonobese	*P* value	Obese	Nonobese	*P* value
*N*	4	118	–	12	242	–
Age (years)	22.3 ± 1.7	22.6 ± 1.3	0.648	27.3 ± 1.4	26.8 ± 1.4	0.198
Body mass index (kg/m^2^)	27.8 ± 2.0	19.6 ± 1.9	0.000	26.0 ± 0.9	20.3 ± 2.0	0.000
Systolic BP (mm Hg)	108.3 ± 3.6	107.5 ± 11.2	0.895	111.2 ± 10.1	106.1 ± 10.7	0.106
Diastolic BP (mmHg)	70.3 ± 4.6	63.9 ± 7.4	0.724	71.5 ± 8.7	65.0 ± 7.8	0.290
Proteinuria (%)	0	1.6	0.000	8.3	1.2	0.136
Serum creatinine (μmol/L)	53.0 ± 7.0	52.7 ± 6.6	0.929	51.4 ± 6.5	52.4 ± 7.1	0.629
eGFR (ml/min)	134.9 ± 16.8	136.2 ± 20.0	0.898	134.5 ± 19.8	132.6 ± 20.8	0.754
FBG (mmol/L)	5.1 ± 0.2	4.7 ± 0.4	0.034	5.0 ± 0.3	4.7 ± 0.4	0.043
TC (mmol/L)	4.92 ± 0.97	4.43 ± 0.69	0.167	4.29 ± 0.86	4.56 ± 0.87	0.289
TG (mmol/L)	1.26 ± 1.07	0.77 ± 0.30	0.005	1.00 ± 0.36	0.83 ± 0.51	0.283
LDL (mmol/L)	2.89 ± 0.84	2.30 ± 0.51	0.029	2.41 ± 0.75	2.39 ± 0.74	0.930
HDL (mmol/L)	1.35 ± 0.29	1.63 ± 0.34	0.114	1.33 ± 0.29	1.59 ± 0.32	0.008
UA (mmol/L)	310.5 ± 36.0	287.2 ± 45.0	0.309	299.6 ± 48.9	277.5 ± 50.8	0.255

Characteristics	30–34 years			35–39 years		
	Obese	Nonobese	*P* value	Obese	Nonobese	*P* value
*N*	16	254	–	43	312	–
Age (years)	32.3 ± 1.2	32.2 ± 1.3	0.698	37.0 ± 1.5	37.0 ± 1.4	0.973
Body mass index (kg/m^2^)	27.5 ± 2.1	20.6 ± 1.9	0.000	26.9 ± 2.0	21.8 ± 2.5	0.000
Systolic BP (mm Hg)	118.4 ± 19.0	105.8 ± 11.6	0.000	109.8 ± 12.5	106.6 ± 12.4	0.107
Diastolic BP (mmHg)	80.3 ± 11.3	69.5 ± 9.3	0.000	74.0 ± 9.9	70.4 ± 8.6	0.064
Proteinuria (%)	6.3	2.0	0.309	2.3	1.2	0.515
Serum creatinine (μmol/L)	50.1 ± 6.5	50.3 ± 7.1	0.889	51.6 ± 7.6	51.8 ± 7.7	0.904
eGFR (ml/min)	134.3 ± 20.6	134.2 ± 22.3	0.994	127.0 ± 21.8	126.4 ± 21.9	0.931
FBG (mmol/L)	5.0 ± 0.5	4.8 ± 0.4	0.159	4.9 ± 0.5	4.9 ± 0.4	0.724
TC (mmol/L)	5.34 ± 1.44	4.56 ± 0.81	0.000	4.86 ± 0.82	4.70 ± 0.77	0.191
TG (mmol/L)	1.45 ± 0.63	0.84 ± 0.34	0.000	1.23 ± 0.63	0.93 ± 0.45	0.000
LDL (mmol/L)	3.26 ± 1.38	2.38 ± 0.65	0.000	2.70 ± 0.76	2.51 ± 0.62	0.083
HDL (mmol/L)	1.42 ± 0.33	1.60 ± 0.34	0.029	1.45 ± 0.26	1.61 ± 0.35	0.003
UA (mmol/L)	309.6 ± 48.1	260.8 ± 49.6	0.000	299.3 ± 51.1	265.7 ± 53.0	0.000

### Risk factors of CKD

**Fig. 1:**
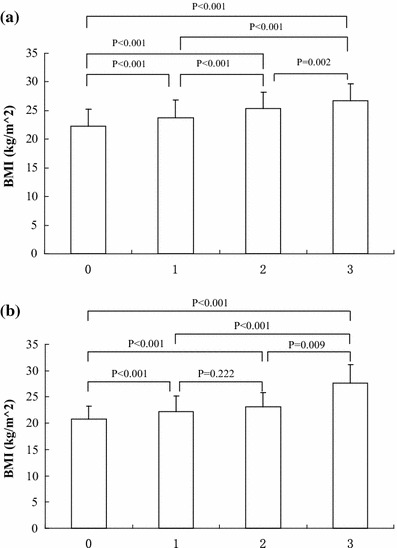
**a** Relationship between male BMI and the number of risk factors. **b** Relationship between female BMI and the number of risk factors

**Fig. 2:**
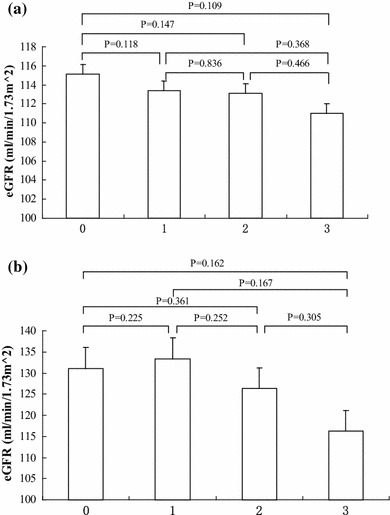
**a** Relationship between male eGFR and the number of risk factors. **b** Relationship between female eGFR and the number of risk factors

We evaluated the relationships between CKD risk factors and BMI, and between the risk factors and eGFR. The percentage of group 0, 1, 2 and 3 in male subjects was 41.6 % (*n* = 538), 37.2 % (*n* = 481), 16.9 % (*n* = 218), and 4.3 % (*n* = 55). The corresponding percentage in female subjects was 82.6 % (*n* = 826), 15.4 % (*n* = 154), 1.7 % (*n* = 17) and 0.4 % (*n* = 4). The mean ages of the four groups in male subjects were 31.4 ± 5.4, 31.7 ± 5.4, 32.8 ± 5.0 and 33.7 ± 4.7, respectively. The mean ages of the four groups in female subjects were 31.2 ± 5.3, 32.0 ± 5.2, 33.2 ± 4.7 and 36.5 ± 3.3, respectively. There was no significant age difference in male and female subjects among all four groups (*P* > 0.05). The mean BMIs of the four groups in male subjects were 22.2 ± 3.0, 23.7 ± 3.1, 25.3 ± 2.9 and 26.7 ± 3.0, respectively. In female subjects, the mean BMIs were 20.8 ± 2.4, 22.2 ± 3.0, 23.1 ± 2.7 and 27.7 ± 3.4, respectively. BMI increased as the number of risk factors increased (Fig. 1). Multivariate analysis indicated that these risk factors were independently associated with obesity, as shown in Table 4. The eGFRs in the four groups were 115.1 ± 17.5, 113.4 ± 17.6, 113.1 ± 17.7 and 111.0 ± 21.2 ml/min in male subjects, and 131.1 ± 21.2, 133.3 ± 24.5, 126.3 ± 17.4 and 116.2 ± 17.0 ml/min in female subjects, respectively. There was no significant difference within group (*P* > 0.05). However, eGFR decreased as the number of risk factors increased (Fig. 2).

**Table 4:** Odds ratios of the risk factors and obesity

	Odds ratio	95 % CI	*P* value
Gender	0.298	0.225–0.395	<0.001
Age	1.039	1.017–1.062	0.001
High BP	2.200	1.694–2.857	<0.001
Hyperglycemia	2.457	1.300–4.644	0.006
Hyperuricemia	1.901	1.467–2.465	<0.001
Dyslipidemia	3.410	2.675-4.347	<0.001

### Multivariate analysis of eGFR

Multivariate analysis showed that in both male and female subjects, eGFR was negatively correlated with age, BMI, UA and TC, positively correlated with FBG, HDL-C, LDL-C and TG, and not correlated with SBP (Table 5). The male and female eGFRs were considered as dependent variables and the age, BMI, DBP, FBG, HDL-C, LDL-C, TC, TG and UA were considered as independent variables in multiple regression analysis. The analysis indicated that in the male group, eGFR was correlated with age, UA, BMI, FBG, TC, TG, LDL-C and HDL-C. In the female group, eGFR was correlated with UA, age, BMI, DBP, FBG and LDL-C (Tables 6, 7).

**Table 5:** Correlation analysis of male and female eGFR and related variables

Group	Age	BMI	SBP	DBP	FBG	HDL	LDL	TC	TG	UA
Male										
*r* value	−0.147	−0.070	0.048	−0.051	0.091	0.058	0.082	−0.100	0.095	−0.124
*P* value	<0.001	0.012	0.089	0.066	0.001	0.039	0.003	<0.001	0.001	<0.001
Female										
*r* value	−0.145	−0.040	0.016	−0.055	0.057	0.080	0.051	−0.071	0.075	−0.298
*P* value	<0.001	0.049	0.457	0.009	0.006	<0.001	0.014	0.001	<0.001	<0.001

**Table 6:** Multiple regression analysis of male eGFR and variables

Variable	Partial regression coefficient	Standard error of mean	Standard partial regression coefficient	*t* value	*P* value
Age	−0.441	0.092	−0.132	−4.778	<0.001
UA	−0.033	0.007	−0.128	−4.514	<0.001
BMI	−0.487	0.175	−0.090	−2.790	0.005
FBG	2.340	0.679	0.095	3.446	0.001
TC	−7.014	1.875	−0.395	−3.740	<0.001
TG	2.566	0.739	0.150	3.472	0.001
LDL-C	5.902	1.947	0.252	3.031	0.002
HDL	5.833	2.754	0.092	2.156	0.031

**Table 7:** Multiple regression analysis of female eGFR and variables

Variable	Partial regression coefficient	Standard error of mean	Standard partial regression coefficient	*t* value	*P* value
Age	−0.560	0.079	−0.139	−7.073	<0.001
UA	−0.084	0.005	−0.329	−15.568	<0.001
BMI	−0.345	0.152	−0.052	−2.272	0.023
DBP	−0.121	0.046	−0.055	−2.635	0.008
FBG	1.854	0.683	0.054	2.716	0.007
LDL-C	−1.170	0.592	−0.041	−1.976	0.048

### Proteinuria

The prevalence of proteinuria in male and female subjects was 2.5 and 1.7 %, respectively, with no significant difference between two groups. The univariate odds ratios for proteinuria of potential risk factors such as BMI, obesity, hypertension, dyslipidemia, high FBG and hyperuricemia are shown in Table 8. The multivariate odds ratios for proteinuria of potential risk factors such as obesity, hypertension, dyslipidemia, high FBG and hyperuricemia are shown in Table 9. Univariate analysis and multivariate analysis showed that obesity, hypertension, dyslipidemia, high FBG and hyperuricemia were independently correlated with proteinuria. Hyperglycemia possesses the highest odds ratios for proteinuria. Conversely, increasing age was independently correlated with lower risk of proteinuria.

**Table 8:** Univariate odds ratios of the risk factors for proteinuria

Risk factor	Odds ratio	95 % CI	*P* value
Gender	0.277	0.122–0.631	0.002
Age	0.977	0.922–1.036	0.442
BMI	1.120	1.034–1.214	0.006
Obesity	2.789	1.470–5.294	0.002
High BP	2.256	1.133–4.494	0.021
Hyperglycemia	13.591	5.897–31.327	<0.001
Hyperuricemia	3.751	1.973–7.131	<0.001
Dyslipidemia	2.781	1.457–5.308	0.002

**Table 9:** Multivariate odds ratios of the risk factors for proteinuria

Risk factor	Odds ratio	95 % CI	*P* value
Gender	0.544	0.215–1.375	0.198
Age	0.952	0.895–1.012	0.114
BMI	0.945	0.799–1.117	0.506
Obesity	1.693	0.545–5.259	0.033
High BP	1.348	0.632–2.874	0.040
Hyperglycemia	10.926	4.413–27.050	<0.001
Hyperuricemia	2.751	1.363–5.550	0.005
Dyslipidemia	1.713	0.802–3.658	0.034

## Discussion

In 2002, a survey conducted by China National Nutrition and Health showed that Chinese adults’ overweight rate was 22.8 %. In our study population, the obesity rate of males between 20 and 39 years old was 33.3 % and of females 7.5 % with an average of 22.0 % which was comparable to the previous finding. Blood pressure, serum uric acid, serum lipids and fasting glucose of obese subjects were higher than those of nonobese subjects. HDL-C of obese subjects was lower than that of nonobese subjects. It indicated that obese young adults had higher risk for developing hypertension, hyperlipidemia, hyperglycemia and hyperuricemia than nonobese counterparts. Hypertension, hyperlipidemia, hyperglycemia and hyperuricemia are the risk factors for CKD. Hypertension has been shown to be a significant risk factor for kidney failure in several large population-based studies [3–5]. Several cohort studies in the USA [6, 7] and Japan [8] have identified hyperlipidemia as also a possible risk factor for the development and progression of CKD. Diabetes has been considered as an initiation risk factor of CKD [9]. Cain et al. [10] found that increasing serum uric acid levels were positively associated with CKD. This association appeared to be independent of age, gender, smoking status, alcohol intake, education, diabetes mellitus, hypertension, BMI and total cholesterol levels. Our study also identified that eGFR decreased as the number of the above risk factors increased.

Obesity may be associated with increased risk for CKD independent of diabetes and hypertension [11]. For example, higher level of proteinuria is a risk factor of CKD [9]. Several studies had indicated that obesity was associated with proteinuria. Chagnac et al. [12] found that non-diabetic obese patients with normal blood pressure had higher albumin excretion rate, higher GFR and glomerular transmembrane pressure than normal people. The rise of glomerular transmembrane pressure will lead to the rise of GRF. The rise of GFR will cause the damage of glomerular filtration membrane and result in the rise of albumin excretion [13]. Tran et al. [14] reported a case of a 48-year-old male patient with severe obesity. When the patient’s weight reduced to 118, 110 and 102 kg, respectively, his 24-h urinary protein excretion reduced to 7.6, 2.1 and 0.85 g, respectively. The CARDIA study [15] showed that an unhealthy lifestyle (unhealthy diet, smoking and obesity) may lead to incident microalbuminuria. But there are few studies on the relationship between obesity and proteinuria in youth. In China, such study has not been performed previously. Our study showed that obesity was an independent risk factor for proteinuria in youth.

There were some limitations associated with our study. First, the subjects might have physiological proteinuria, but they had no fever and had been told not to do strenuous exercise before the test, which could decrease the false-positive rate. Second, we did not test microalbuminuria. So, the morbidity of proteinuria might be underestimated in our study. Third, the diagnosis of obesity was based on the standard of Chinese Diabetes Society. We did not measure waists and hips. Thus, visceral obesity might be missed, which could also decrease the false-positive rate. Fourth, we used the equation of MDRD to estimate the glomerular filtration rate (eGFR). The MDRD equation was first proposed by the National Kidney Foundation, K/DOQI clinical practice guidelines for chronic kidney disease: evaluation, classification, and stratification in 2002 [2]. The MDRD equation is now widely used in nephrology. But this equation has some limitations. For example, creatinine is affected by the muscle mass of the subject. So if the subject is too fat or too thin, this equation is not accurate enough to estimate the GFR. Serum cystatin C has been proposed as a simple, accurate and rapid endogenous marker of GFR in research and clinical practice. It is more stable than creatinine and less influenced by the muscle and gender. One meta-analysis showed that serum cystatin C was clearly superior to serum creatinine as a marker of GFR [16]. So in our further study, we may ues cystatin C to estimate GFR.
